# Unheeded SARS-CoV-2 proteins? A deep look into negative-sense RNA

**DOI:** 10.1093/bib/bbac045

**Published:** 2022-03-01

**Authors:** Martin Bartas, Adriana Volná, Christopher A Beaudoin, Ebbe Toftgaard Poulsen, Jiří Červeň, Václav Brázda, Vladimír Špunda, Tom L Blundell, Petr Pečinka

**Affiliations:** 1 Department of Biology and Ecology, University of Ostrava, Ostrava 710 00, Czech Republic; 2 Department of Physics, University of Ostrava, Ostrava 710 00, Czech Republic; 3 Department of Biochemistry, Sanger Building, University of Cambridge, Tennis Court Rd, Cambridge CB2 1GA, UK; 4 Department of Molecular Biology and Genetics, Aarhus University, 8000 Aarhus, Denmark; 5 Institute of Biophysics, Czech Academy of Sciences, Brno, 612 65, Czech Republic; 6 Global Change Research Institute, Czech Academy of Sciences, Brno, 603 00, Czech Republic

**Keywords:** SARS-CoV-2, RNA, ORFs, Kozak sequence, proteomics, structures

## Abstract

SARS-CoV-2 is a novel positive-sense single-stranded RNA virus from the *Coronaviridae* family (genus *Betacoronavirus*), which has been established as causing the COVID-19 pandemic. The genome of SARS-CoV-2 is one of the largest among known RNA viruses, comprising of at least 26 known protein-coding loci. Studies thus far have outlined the coding capacity of the positive-sense strand of the SARS-CoV-2 genome, which can be used directly for protein translation. However, it has been recently shown that transcribed negative-sense viral RNA intermediates that arise during viral genome replication from positive-sense viruses can also code for proteins. No studies have yet explored the potential for negative-sense SARS-CoV-2 RNA intermediates to contain protein-coding loci. Thus, using sequence and structure-based bioinformatics methodologies, we have investigated the presence and validity of putative negative-sense ORFs (nsORFs) in the SARS-CoV-2 genome. Nine nsORFs were discovered to contain strong eukaryotic translation initiation signals and high codon adaptability scores, and several of the nsORFs were predicted to interact with RNA-binding proteins. Evolutionary conservation analyses indicated that some of the nsORFs are deeply conserved among related coronaviruses. Three-dimensional protein modeling revealed the presence of higher order folding among all putative SARS-CoV-2 nsORFs, and subsequent structural mimicry analyses suggest similarity of the nsORFs to DNA/RNA-binding proteins and proteins involved in immune signaling pathways. Altogether, these results suggest the potential existence of still undescribed SARS-CoV-2 proteins, which may play an important role in the viral lifecycle and COVID-19 pathogenesis.

## Introduction

The Severe Acute Respiratory Syndrome Coronavirus-2 (SARS-CoV-2) has been intensively studied worldwide for its role as the causative agent of the COVID-19 pandemic [1–3]. Coronaviruses, such as SARS-CoV-2, are single-stranded positive-sense RNA viruses and have the largest genomes among RNA viruses—usually around 30 kb. It has been established that the SARS-CoV-2 genome codes for at least 26 proteins: 16 nonstructural proteins (NSP1-16), 4 structural proteins (surface glycoprotein, membrane glycoprotein, envelope protein and nucleocapsid phosphoprotein) and 6–9 accessory factors (designated as open reading frames, ORFs) [4–7]. The nonstructural proteins are all encoded among the ORF1ab gene, which is comprised of two smaller ORFs, ORF1a (nsp 1-11) and ORF1b (nsp 12-16), that are separated by a −1 ribosomal slippage event [8]. The ORF1ab gene is followed by the genes coding for the structural and accessory proteins, among which several overlapping genes and new ORFs, which may code for new proteins, have been discovered in the accessory region in recent months as well [9]. Many of these accessory ORFs, such as ORF-3d-2 and ORF-Sh, have been only discovered using phylogenomic methodologies, which requires further experimental validation with proteomics or ribosome profiling techniques [10]. Altogether, these studies suggest that the SARS-CoV-2 proteome has not been completely resolved.

Positive-sense RNA viruses, such as SARS-CoV-2, have been thought to encode proteins solely on the positive strand. However, Dinan *et al.* [11] recently demonstrated that negative-sense viral RNA strand intermediates arising during replication of viral positive-sense RNA genomes also have protein-coding potential. Previous Ribo-Seq analysis of an infection model with the murine coronavirus (mouse hepatitis virus, strain A59) revealed that negative-sense RNA was found at significantly lower levels than the positive-sense and that translation on the negative strand was uncertain [12]. One study looking at the conservation of protein-coding genes among the SARS-CoV-2 and other related coronavirus genomes extended their search to the negative strand and found no convincing results [13]. Although studies have quantified the amount of SARS-CoV-2 negative-sense RNA in host cells, which is present at approximately 10–100 times lower than positive-sense RNA, no studies to date have described the potential for coding sequences on the negative strand of the SARS-CoV-2 genome [14].

Herein, a combination of complementary sequence and structure-based bioinformatic approaches was used to elucidate the presence of protein-coding negative-sense ORFs (nsORFS) in the SARS-CoV-2 genome. First, we identified and cross-examined the presence of eukaryotic translation initiation sites [15, 16] and ORFs on the SARS-CoV-2 negative-sense genome using four distinct tools. The predicted nsORFs were then subjected to codon bias analysis, transcription factor binding site analysis, sequence and domain-based homology searches, proteomic meta-searches, ribosome profiling analysis and 3D structure prediction and characterization to understand their potential validity and functionality. In summary, we discovered nine putative protein-coding regions on the negative-sense SARS-CoV-2 RNA that exhibited codon biases consistent with the human genome and were predicted to contain higher-order 3D structural folding. We extended our reach to check for nsORFs in phylogenetically related coronavirus genomes and discovered that the presence of protein-coding regions on negative-sense coronavirus RNA may be evolutionarily conserved and widespread. Proteomics and Ribo-Seq analyses were unable to detect whether these nsORFs are translated during infection; however, because of the low amount of negative-sense RNA, detection of translation may require more focused and in-depth experimentation. Our analyses propose novel SARS-CoV-2 ORFs that may play a role during infection of host cells.

## Results and discussion

### Novel ORFs with Kozak consensus sequences detected on SARS-CoV-2 negative-sense strand

The detection of translation initiation sequences in viral genomes for the prediction and characterization of potential protein-coding sequences has been described for several viral pathogens [17–20]. In order to detect potential coding sequences on the SARS-CoV-2 negative-sense genome, we used two web servers, TISrover [21] and ATGpr [22], that detect eukaryotic ribosome translation initiation sites (TIS) based on machine learning algorithms and two web servers, NCBI ORFfinder (https://www.ncbi.nlm.nih.gov/orffinder/) and StarORF (http://star.mit.edu/orf/index.html), that look for ORFs based on six-frame translation of nucleotide sequences. The TIS detection tools search for eukaryotic translation start signals, such as the Kozak sequence (A/GXXATGG), which have been recorded to significantly affect gene expression [23, 24]. The TIS detection tools provide confidence scores from 0 to 1 that can be used to discern the probability of the predicted start site. The SARS-CoV-2 positive strand and its recorded gene start sites were analyzed in parallel using the TIS detection tools as a control measure and to set threshold values for the TIS detection tools [3, 9]. The TIS detection tools correlated well with the positive strand gene start sites (Supplementary Table S1). The first eight start sites found using ATGpr corresponded to the M, ORF9b, N, ORF1ab, ORF8, truncated version of N, and S genes, while also detecting the ORF3a, ORF7a and ORF9c genes above the 0.1 score. TISrover presented lower sensitivity but still detected the M, N, ORF7b, ORF1ab, ORF6, ORF8 and ORF3a start sites at above a 0.1 score. We, thus, set a threshold value of 0.1 for both ATGpr and TISrover for detection of putative TIS sites on the negative strand. A value of 0.1 has also been established as a threshold using ATGpr in other human and viral TIS detection studies [25, 26]. Three criteria were established for selection of potential ORFs: the sequences must be (1) found using all four tools or (2) found in both TIS detection tools above the 0.1 threshold and (3) sequence length must be above 40 amino acids. After filtering based on the criteria, nine sequences were selected to be potentially protein-coding on the negative strand of SARS-CoV-2. Each of the nine had a strong Kozak signal, a stop codon and ranged from 132-300 nt (Table 1). Corresponding nucleotide and amino acid sequences are enclosed in Supplementary files 1 and 2 in FASTA format.

**Table 1:** Sequence position and analysis of nsORFs

nsORF	Frame	Identity to Kozak rule (A/GXXATGG)	Start (bp)	Finish (bp)	Length (nuc/aa)	ATGpr score	TISrover score	CAI
nsORF1	2	AXXATGc	562	694	132 / 44	0.1	0.861	0.717
nsORF2	2	tXXATGt	2899	3097	198 / 66	0.06	0.008	0.712
nsORF3	3	cXXATGa	5792	5975	183 / 61	0.09	0.028	0.693
nsORF4	2	tXXATGa	6466	6703	237 / 79	0.16	0.102	0.806
nsORF5	1	AXXATGa	8865	9057	192 / 64	0.09	0.194	0.654
nsORF6	1	GXXATGt	10 047	10 188	141 / 47	0.11	0.909	0.739
nsORF7	3	AXXATGt	23 414	23 714	300 / 100	0.22	0.015	0.682
nsORF8	1	cXXATGa	29 211	29 385	174 / 58	0.14	0.232	0.705
nsORF9	2	AXXATGG	29 236	29 479	243 / 81	0.47	0.889	0.776

**Figure 1:**
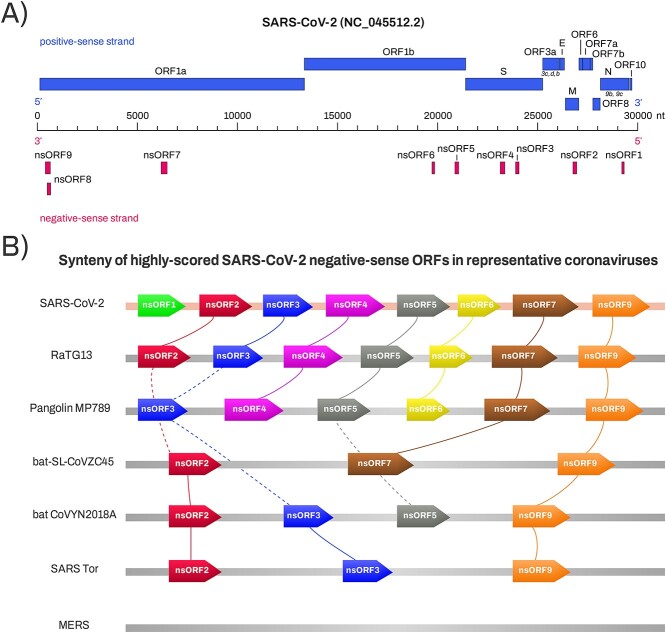
Localization and synteny of nsORFs in SARS-CoV-2 and related coronaviruses. (**A**) Localization of all identified nsORFs within the SARS-CoV-2 genome. The upper part of the scheme depicts positively encoded ORFs annotated on NCBI reference SARS-CoV-2 genome, together with additional ORFs described in the literature (indicated in italics): ORF3c, ORF3d, ORF3b (which span particular genomic regions of ORF3a) and ORF9b and ORF9c (which span particular genomic regions of N). (**B**)Synteny of SARS-CoV-2 nsORFs in representative species of SARS-like coronaviruses. At least two SARS-CoV-2 nsORFs (nsORF2 and nsORF9) are more or less conserved in most of inspected SARS-CoV-2-related coronaviruses, including old SARS-CoV Tor 2003. In MERS-CoV and human-CoV-OC43, none of SARS-CoV-2 homologous nsORFs was found. The synteny plot was constructed using SimpleSynteny web server [56] and redrawn in this schematic figure.

The predicted negative-sense ORFs (nsORFs) were numbered in order of their appearance in the 5′ → 3′ direction of the negative-sense RNA (nsORF1-9). The putative nsORFs are found distributed throughout the negative-sense strand and two, nsORF8 and nsORF9, are overlapping on different reading frames. Based on the 5′ → 3′ directionality of the positive strand, 5 of the nsORFs are found within the ORF1ab region, and the remaining 4 are found among the structural and accessory protein genomic regions (Figure 1). Amino acid sequence-based similarity detection tools (Pfam, SMART and CDD search) were unable to detect homologous genes for all predicted nsORFs.

To explore whether predicted nsORFs contain binding motifs for human proteins, further bioinformatic analysis using the Beam RNA Interaction motif search tool [27] was conducted (Supplementary Table S3). This RNA interaction motif analysis revealed many interesting hits: nsORF9 (and its overlapping nsORF8) contains a motif that is significantly similar to the PUM1 binding sequence (*P*-value = 0.012). The PUM1 protein has been reported to play a role in cytoplasmic sensing of viral infection [28]. nsORF7 contains a motif that is significantly similar to the UPF1 binding sequence (*P*-value = 0.019); this protein is also called the regulator of nonsense transcripts 1 and plays a vital role in host–virus interaction [29]. nsORF6 contains a sequence motif that is significantly similar to the MOV10 binding sequence (*P-*value = 0.0083), and MOV10 has been identified to exhibit antiviral activity against dengue virus (which is also a positive-sense ssRNA virus) [30]. Interestingly, MOV10 is a ‘5′ to 3′ RNA helicase contributing to UPF1 mRNA target degradation by translocation along 3′ UTRs’ [31]. nsORF5 contains a motif that is significantly similar to the ATP-dependent RNA helicase SUPV3L1 binding sequence (*P-*value = 0.012); as this protein is considered to be mitochondrial [32], the interaction with SARS-CoV-2 RNA seems to be unlikely. nsORF4 contains a motif that is significantly similar to the heterogeneous nuclear ribonucleoprotein L (hnRNP L) binding sequence (*P-*value = 0.02). Notably, it was previously reported that hnRNP L interacts with hepatitis C virus (positive-sense ssRNA virus) 5′-terminal untranslated RNA and promotes efficient replication [33]. nsORF3 contains a motif that is significantly similar to the U2AF5 binding sequence (*P*-value = 0.0091) and also a motif that is significantly similar to the hnRNP L binding sequence (*P*-value = 0.025), as in nsORF4. nsORF2 contains a motif that is significantly similar to the GRWD1-binding sequence (*P*-value = 0.00022), but the functions of this protein are still largely unknown. nsORF1 contains motifs that are significantly similar to nuclear cap-binding protein subunit 3 (NCBP3) binding sequence (*P*-value = 0.034) and U2AF2 binding sequence (*P*-value = 0.04). NCBP3 associates with NCBP1/CBP80 to form an alternative cap-binding complex which plays a key role in mRNA export; it is also known that the alternative cap-binding complex is important in cellular stress conditions such as virus infections and the NCBP3 activity inhibit virus growth [34].

In addition, we revealed that approximately half of identified proteins that are predicted to bind nsORFs RNA are linked to the FMR signaling pathway [35], which could perhaps partially explain the diverse repertoire of brain-related symptoms, that is the frequently mentioned ‘brain fog,’ increase of depression and other mental issues in post-COVID patients [36, 37]. We also revealed specific transcription factors that may bind to nsORFs RNA, for example ZNF622 and ZNF800 (binding sites within nsORF9, nsORF8 and nsORF6), which further supports our hypothesis about the possible expression of such nsORFs. STRING analysis [38] of all proteins predicted to interact with nsORFs RNA revealed significant enrichment of several molecular and biological processes, such as alternative splicing, RNA processing, gene silencing and so on. (Supplementary Table S2), which could potentially explain heterogenous and unexpectable symptoms of COVID-19 patients—from the muscle pain [39] to hepatobiliary and pancreatic injury [40]. All mentioned symptoms are frequently explained by a decrease of oxygen saturation or inflammatory factors, but, herein, we may have further demystified the complex mosaic of signaling behind such manifestations.

Codon usage similarity between viral and host genomes has been shown as an indicator for adaptation to the host as optimized use of the available endogenous amino acids allows more efficient translation of viral genes [41, 42]. The codon usage of the canonical SARS-CoV-2 genes has been determined to correlate well with the human, bat and pangolin amino acid pools [43, 44]. Using the codon adaptability index (CAI), which has been shown as an accurate predictor for gene expression levels, studies have shown that the CAI values of the positive strand average around 0.7 (with 1 being the best score) [45–47]. Thus, to better understand the expression efficiency of the putative nsORFs and their codon usage similarity to the human amino acid pool, codon usage tables were created and analyzed using COUSIN and the CAI values for the nsORFS were calculated using the CAIcal web server [48, 49]. Comparison of the relative frequencies of codons used by the positive and negative-sense genomes in relation to the human genome revealed a high correlation between preferred codons. As shown in Table 1, average CAI values for the positive strand were 0.68 and ranged from 0.606 to 0.726, while the average for negative strand ORFs was 0.72 and ranged from 0.654 to 0.806. Notably, nsORF4, nsORF6 and nsORF9 reported higher CAI values (0.806, 0.739 and 0.776 respectively) than the maximum reported CAI for the positive strand genes (N protein: 0.726). The high congruence between the CAI values of the negative and positive-sense ORFs to the human amino acid pool lends further evidence for potential expression of these genes.

In order to detect whether the nsORFs are translated in human cells, we performed (1) proteomics meta-searches of the mass spectrometry data from two other studies involving SARS-CoV-2 infection of human primary alveolar macrophages [50] and Vero E6 cells [51]; and (2) an analysis of ribosomal profiling data from Finkel et al. [7]. Unfortunately, the signals were too weak in both cases to confirm translation. Studies have shown that lowly expressed proteins, such as the E protein (only 20 copies per virion [52]), may not be discovered using proteomics techniques [53, 54]. Additionally, negative-sense RNA has been to present at 10–100 times lower than the amount of positive-sense RNA [14]. The Ribo-Seq data reflected this pattern, as the gene transcript mapping failed to attain a threshold level of genome coverage [9]. Thus, more focused or high-depth Ribo-Seq profiling or proteomics may better resolve the *in vivo* presence of these proteins.

### Evolutionary conservation of nsORFs

Evolutionary conservation of ORFs has been considered as supporting evidence for protein expression [55]. Thus, we used SimpleSynteny tool [56] to investigate nsORF synteny among coronaviruses. We have found, that in the closest relative, RaTG13 coronavirus genome, nearly all nsORFs (except of nsORF1) are conserved and not truncated by stop codons (Figure 1). In more distant coronaviruses (e.g. bat SARS-like coronavirus isolate bat-SL-CoVZC45, coronavirus BtRs-BetaCoV/YN2018A and SARS coronavirus Tor2), 3–4 SARS-CoV-2 nsORFs still have their homologs (Figure 1). Interestingly, nsORF3 and nsORF5 were truncated in bat SARS-like coronavirus isolate bat-SL-CoVZC45, but preserved in evolutionarily more distant coronavirus BetaCoV/YN2018A. nsORF2 and nsORF9 were conserved in all inspected viral strains of SARS-related coronaviruses, and these are SARS-CoV-2 nsORFs identified by all four approaches—TISrover, ATGpr, NCBI ORFfinder and StarORF [57]. In MERS-CoV, human-CoV-OC43, and more distant members of *Coronaviridae* family, no homologous SARS-CoV-2 nsORFs were found.

### ORFs predicted to contain higher order folding: modeling, characterization and comparison

**Figure 2:**
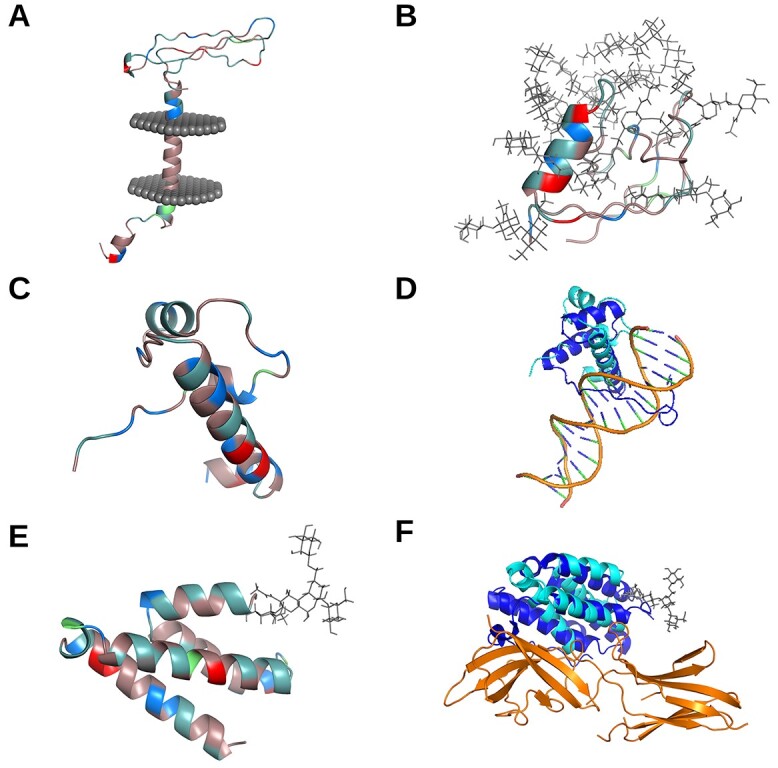
Structural characterization and similarity comparisons of nsORFs. Residues of putative nsORFs in A (nsORF7), B (nsORF5), C (nsORF1) and E (nsORF9) are depicted with amino acid colouration: red for acidic (D and E), blue for basic (H, R and K), light teal for polar noncharged (S, N, T and Q), dirty violet for hydrophobic (A, V, I, L, M, F, W, P, G and Y), and lime green for cysteine residues. Putative transmembrane protein nsORF7 is shown with the predicted transmembrane region inside a representative cell membrane (**A**). Extensive O-linked glycosylation of nsORF5 is shown with gray stick configurations (**B**). The structural similarity of nsORF1 (**C**) to a homologous protein of T-cell leukemia homeobox protein 2 (which was predicted by RUPEE, but shown without DNA) bound to DNA (PDB: 3a01; both homeobox protein structures are published by [76]) is depicted with nsORF1 in cyan, homeobox protein in blue, and DNA in orange (**D**). The predicted protein–protein interaction of nsORF9 (**E**) and interferon alpha/beta receptor 2 using HMI-PRED is compared to the interaction between interferon alpha/beta receptor 2 and interferon omega-1 (PDB: 3se4) with nsORF9 in cyan, interferon omega-1 in blue, and interferon alpha/beta receptor 2 in orange (**F**).

To gain more insight into the potential functionality of these genes, despite the uncertainty of their translation, we predicted the 3D structure of each nsORF, characterized the predicted structures and performed structural comparisons with all 3D experimentally resolved proteins. As no templates were available for homology modeling, *ab initio* structural modeling with trRosetta [58] was used in combination with secondary structure prediction and structural refinement with RaptorX [59] and MODELLER [60, 61], respectively. All nsORFs were predicted to have higher order folding (Figure 2). Potential transmembrane region analysis using TMHMM and the OPM database revealed that only nsORF7 was predicted to contain a transmembrane domain (Figure 2A) [62, 63]. To study the effect of major post-translational modifications, N- and O-linked glycosylation motifs were detected using NetNGlyc [64] and NetOGlyc [65] and modeled using the CHARM-GUI Glycan Reader and Modeler [66]. nsORF9 and nsORF6 were predicted to have one and two N-linked glycosylation sites, respectively, and nsORF5 and nsORF3 were predicted to contain 10 and 4 O-linked glycosylation sites, respectively (Figure 2B). Heavy glycosylation may imply potential roles in inflammatory processes as secreted signaling proteins [67]. Isoelectric points, predicted by ExPASy [68], were found at an average 9.07, which is reflected by the higher presence of basic residues, as shown in Figure 2C. The presence of positively charged residues may have implications in viral or host nucleic acid binding [69, 70]. Overall, the nsORFs were found to contain higher order folding and several structural characteristics of interest.

Structural similarity comparisons have been shown to give insight into potential protein–protein interactions, despite low sequence similarity [71, 72]. Using RUPEE [73], the nsORFs were compared to all known protein families, and HMI-PRED [74] was used to infer potential host interaction partners. Structural alignments generated using RUPEE revealed that three nsORFs, 2, 4 and 9, exhibited high structural similarity to known proteins with TM-scores over 0.5 (indicating that they are in the same fold), while nsORFs 1, 3, 5 and 8 reported the lowest similarity with TM-scores under 0.4 [75]. The highest returned TM scores of the nsORFs, such as 1, 4 and 9, were predicted to be structurally similar to RNA/DNA binding proteins (T-cell leukemia homeobox protein 2, DNA-binding domain of mouse MafG and RNA-binding domain from influenza virus nonstructural protein 1, respectively), furthering evidence from the isoelectric point observations (Figure 2D) [76]. Cell signaling factors, such as those involved in complement activation, may be mimicked by nsORF6 and nsORF8, while proteins involved in protein degradation and other ubiquitin-related processes might interact with nsORF2, nsORF3, nsORF6 and nsORF9 (Table 2). Interestingly, only nsORFs 2, 4, 7 and 9 returned potential mimicked/disrupted protein–protein interfaces by HMI-PRED (Supplementary Table S3). Diverse cellular processes were predicted to be involved in the mimicked interfaces; for instance, nsORF9 was found structurally similar to interferon alpha/beta receptor 2 binding to interferon omega-1, which could have roles in inflammatory signaling in SARS-CoV-2 infections (Figure 2F) [77]. The higher order folding of these ORFs and similarity to known proteins provides further evidence that they may have functional roles in infection.

**Table 2:** Structural characterization of nsORFs

					Selected RUPEE Hits			
nsORF	Isoelectric Point	NetNGlyc Residue #	NetOGlyc Residue #	# HMI-PRED Hits	Superfamily	Structure name	PDB (chain)	TM-score
nsORF1	12			0	Homeodomain-like	T-cell leukemia homeobox protein 2	3a03(a)	0.51
					Histone-fold	Histone h4	4z2m(h)	0.5
					FF domain	Formin-binding protein 3	2cqn(a1)	0.49
nsORF2	9.78		3,8,12,21	16	UBA-like	Ubiquitin carboxyl-terminal hydrolase 5	2dag(a1)	0.39
					Insulin-like	Insulin-like growth factor II	1igl(a)	0.35
nsORF3	8.61		14,29,29,31,32,33,34,44,50	0	RING/U-box	E3 ubiquitin-protein ligase AMFR	2lxp(c)	0.36
					Viral DNA-binding domain	Regulatory protein E2 from human papillomavirus	1f9f(b1)	0.35
nsORF4	9.46			18	A DNA-binding domain in eukaryotic transcription factors	Mouse MafG	1k1v(a)	0.53
					Phosphoprotein XD domain	RNA polymerase alpha from measles virus	2k9d(a)	0.42
nsORF5	11	25,38		0	YegP-like	nmb1088 protein from *Neisseria meningitidis*	3bid(f2)	0.44
					Complement control module/SCR domain	Complement receptor type 1	2mcz(a2)	0.41
					Signal recognition particle (SRP) complex	Signal recognition particle 9 kDa protein	1ry1(c)	0.4
					Scorpion toxin-like	Hongotoxin 1	1hly(a)	0.36
nsORF6	6			0	WW domain	NEDD4-like E3 ubiquitin-protein ligase WWP1	2op7(a)	0.34
					Immunoglobulin	Obscurin	2edf(a1)	0.31
nsORF7	7.71			47	PX domain	Sorting nexin-17	3foga(1)	0.46
					Histone-fold	Histone h4	3nqu(b)	0.45
nsORF8	7.87			0	Bowman-Birk inhibitor, BBI	Bowman–Birk type proteinase inhibitor	2iln(i)	0.38
					Complement control module/SCR domain	Complement control protein from vaccinia virus	1rid(b3)	0.34
nsORF9	9.22	51		21	GAT-like domain	ADP-ribosylation factor-binding protein GGA1	1x79(a)	0.65
					MIT domain	Vacuolar protein sorting-associating protein 4B	2jqh(a)	0.56
					BAG domain	BAG-family molecular chaperone regulator-4	1m62(a1)	0.56
					tRNA-binding arm	*Staphylococcus aureus* femA	1lrz(a)	0.54
					S15/NS1 RNA-binding domain	Nonstructural protein 1 from influenza virus	1 ns1(a)	0.53

## Conclusion

Altogether, our results suggest the existence of still undescribed SARS-CoV-2 proteins, which may play an important role in the viral lifecycle and COVID-19 pathogenesis. Nine potential nsORFs were discovered using various sequence- and structure-based bioinformatic methodologies. The nsORFs were unable to be detected using publicly available proteomics or ribosomal profiling datasets, which may reflect their low overall abundance. Interestingly, the average codon adaptability of the nsORFs was higher than that of the positive-sense SARS-CoV-2 genes, which may be a compensatory mechanism to account for low levels of negative-sense RNA as templates for translation. All nine nsORFs were predicted to have higher order folding, which was confirmed by the structural similarity to several known human and viral proteins. For example, both nsORF2 and nsORF9 were predicted to have histone-like folds. Furthermore, nsORF2 contains sorting nexin-like fold, and nsORF9 contains formin-binding-like fold. Notably, sorting nexin proteins and formin-binding proteins are known to be interaction partners, which may give more indications for their complementary roles during infection [57]. Is it therefore possible that some of the SARS-CoV-2 nsORFs are expressed and form protein complexes similarly as nsp1–nsp16 on the positive SARS-CoV-2 RNA strand? We hope that this study will stimulate further research in the field of developing more specific and sensitive approaches to detect the complete SARS-CoV-2 proteome *in vitro* and *in vivo*.

## Materials and methods

### Sequence collection and ORF detection and characterization

The SARS-CoV-2 reference genome (NC_045512.2) was selected and reverse-transcribed using Reverse complement tool (https://www.bioinformatics.org/sms/rev_comp.html) as a reference for the negative-sense strand. A combination of four tools was used to discover ORFs and Kozak sequences on the negative-sense strand: TISRover prediction tool for predicting translation initiation sites in human by convolutional neural networks [21]; ATGpr tool (https://atgpr.dbcls.jp/) that uses linear discriminant analysis for identifying the initiation codons [22]; NCBI ORFfinder (https://www.ncbi.nlm.nih.gov/orffinder/) to predict all potential ORFs; and StarORF (http://star.mit.edu/orf/index.html) to cross examine ORFfinder results. The Beam RNA Interaction motif search tool (BRIO) [27] was used for the transcription factor binding site analysis with default parameters and all resulting hits are enclosed in the Supplementary Table S2. Interaction network analysis of proteins predicted by BRIO was done using STRING tool [38] with default parameters (https://string-db.org/cgi/input?sessionId=bVBUeCTKWYuE&input_page_show_search=on). Codon usage tables were made using COUSIN [49], and the codon adaptability index (CAI) was calculated using the CAIcal web server [48]. To check for possible domain homologs, we used NCBI’s Conserved Domains Database (CDD) webserver (https://www.ncbi.nlm.nih.gov/Structure/cdd/wrpsb.cgi) [78] with an E-value cut-off set to 10 and cross-validated with Pfam (http://pfam.xfam.org/search#tabview=tab1) [79] and SMART tools (http://smart.embl-heidelberg.de/) [80] (default parameters).

### ORF conservation and synteny in related viral species

To inspect whether there are proteins homologous to the SARS-CoV-2 negatively encoded ORFs also in another important coronaviral species, we made tblastn searches (https://blast.ncbi.nlm.nih.gov/Blast.cgi) using negatively encoded ORFs (protein sequences) as a query. The searches and further analyses were restricted to the representative betacoronaviral (β-CoV) genomes listed in Supplementary Table S4. Synteny was analyzed and graphically depicted using the SimpleSynteny tool (https://www.dveltri.com/simplesynteny/about.html) [56].

### Structural characterization of potential protein-coding sequences

The trRosetta web server (https://yanglab.nankai.edu.cn/trRosetta/) was used [81] for *ab initio* modeling. RaptorX and MODELLER were used for secondary structure predictions and structural refinement, respectively. The resulting structures were visualized with the UCSC Chimera 1.15 workflow [82]. RUPEE was used to perform sequence-independent structural comparisons, and HMI-PRED was utilized to infer host–microbe interactions using structural alignment and protein–protein docking methodologies. To compute Mw and isoelectric point (pI), we used the Expasy Compute pI/Mw tool (https://web.expasy.org/compute_pi/) [68]. N- and O-linked glycosylation were predicted using NetNGlyc (http://www.cbs.dtu.dk/services/NetNGlyc/) [64] and NetOGlyc (http://www.cbs.dtu.dk/services/NetOGlyc/) [65], respectively.

### Translation detection

Assessment of nsORF1-9 expression was performed by researching LC–MS/MS data from two previously published SARS-CoV-2 studies looking at the infection of human alveolar macrophages [50] and green monkey Vero E6 cells [51]. Data were either search against the human or green monkey SWISS-PROT [83] reference proteomes (Human db: 09-2020, 20,609 sequences; Green monkey: 08-2020, 19 229 sequences) and the UniProt [84] SARS-CoV-2 database (12-2020, 16 sequences) to which the putative nsORF1-9 protein sequences had been included. Data were searched using the Mascot search engine (Matrix Science, v.2.5) or by using Proteome Discoverer (Thermo Scientific, v.2.5) employing the Sequest HT and MS Amanda 2.0 search engines. Extended search criteria are included in SM6. Ribosome profiling analyses was performed as described in Ardern *et al.* [9] using data from Finkel et al. [7] (SRR11713356-61, SRR12216748-54).

**Key Points**

- According to our findings, the genome of SARS-CoV-2 contains several negative-sense ORFs.
- These ORFs were validated using the combination of bioinformatic approaches.
- Structural modeling revealed the presence of higher order folding in these putative proteins.
- Structural mimicry analyses suggest similarity to DNA-/RNA-binding proteins and proteins involved in immune signaling pathways.
- Results suggest the potential existence of still undescribed SARS-CoV-2 proteins, which may play an important role in the viral lifecycle and COVID-19 pathogenesis.

## Supplementary Material

Supplementary_table_S1_bbac045

Supplementary_table_S2_bbac045

Supplementary_table_S3_bbac045

Supplementary_table_S4_bbac045

Supplementary_file_1_-_nucleotide_sequences_bbac045

Supplementary_file_2_-_protein_sequences_bbac045

## Notes

**Martin Bartas** is a postdoctoral researcher in the Department of Biology, University of Ostrava, Czech Republic. His research interests include molecular virology, noncanonical forms of nucleic acids, protein interactions and bioinformatics.

**Adriana Volná** is a PhD student in the Department of Physics, University of Ostrava, Czech Republic. Her work spans molecular virology, plant biology and interdisciplinary approaches.

**Christopher A. Beaudoin** is a PhD candidate in the Department of Biochemistry, at the University of Cambridge. His research areas are drug discovery, computational biology and bioinformatics.

**Ebbe Toftgaard Poulsen** is an assistant professor at Aarhus University with a focus on mass spectrometry analyses and research in molecular biology and genetics.

**Jiří Červeň** works as a research fellow in the Department of Biology, University of Ostrava, Czech Republic. His work spans molecular biology and microbiology.

**Václav Brázda** is a professor and the head of the Laboratory of Protein–DNA Interactions, Institute of Biophysics of the Czech Academy of Sciences, Brno, Czech Republic. He is studying the interaction of proteins with DNA, local DNA structures and p53 protein, and is a co-author of a web-bioinformatics server.

**Vladimír Špunda** is an assistant professor and the Head of the Department of Physics, University of Ostrava, Czech Republic. Vladimír does research in biophysics, biochemistry and ecophysiology of photosynthesis.

**Tom L. Blundell** is a professor at the Department of Biochemistry, University of Cambridge. His research focuses on structural biology, bioinformatics and drug discovery for cancer and mycobacterial infections.

**Petr Pečinka** is an assistant professor and the team leader of the molecular biology group in the Department of Biology, University of Ostrava, Czech Republic.
